# The complete mitochondrial genome of Eastern Curlew *Numenius madagascariensis* (Charadriiformes, Scolopacidae)

**DOI:** 10.1080/23802359.2021.1959448

**Published:** 2021-09-30

**Authors:** Tianhui Guo, Zhiyuan Yang, Ya Zhu, Hao Wang, Du Kong, Chaochao Hu, Mingqing Liu

**Affiliations:** a College of Life Sciences, Jiangsu Key Laboratory for Biodiversity and Biotechnology, Nanjing Normal University, Nanjing, China; b Ministry of Ecology and Environment, Nanjing Institute of Environmental Sciences, Nanjing, China; c Nanjing Private Experimental School, Nanjing, China; d Nanjing No.9 Middle School, Nanjing, China

**Keywords:** Mitogenome, *Numenius madagascariensis*, phylogenetic analysis, Scolopacidae

## Abstract

The complete mitochondrial genome of *Numenius madagascariensis* Linnaeus, 1766 was sequenced in this study. The circular mitogenomes was 17,147 bp in length, containing 13 protein-coding genes, two ribosomal RNAs (12S rRNA and 16S rRNA), 22 transfer RNA genes, and a D-loop region. The overall nucleotide composition was A: 31.0%, T: 25.6%, C: 29.5%, and G: 13.9%. Twenty-eight genes were encoded on the heavy strand, while the remaining nine genes were encoded on the light strand. The common start codon was ATG, and three stop codons and an incomplete stop codon (T–) were used in PCGs. This study improves our comprehension of the mitogenomic characteristics and its phylogenetic relationships within Scolopacidae.

The Eastern Curlew *Numenius madagascariensis* is one of the largest migrant shorebirds traveling along the East Asian-Australasian Flyway, which has an extremely large geographic range throughout Australia, eastern and southern Asia (Xiao et al. 2021). It is a large long-billed shorebird with mostly brown in color, differentiated from other curlews by its plain, unpatterned brown underwing. With an extremely rapid population reduction, Eastern Curlew is listed as Endangered (BirdLife International 2017). The reasons for the decline possibly including habitat loss, landscape transformation, change in agricultural and the human induced factors (Schwemmer et al. 2016; Dhanjal-Adams et al. 2017, 2019). To date, the mitochondrial genome sequencing database has rapidly increased in recent years (Sharko et al. 2019), while the basic genetics data of *N. madagascariensis* has not been well studied. In this study, we sequenced and annotated the complete mitogenome of *N. madagascariensis* to explore the mitogenomic characteristics and its phylogenetic relationships within Scolopacidae.

The sample was collected in Chongming, Shanghai, China (31.6395 N, 121.5181 E), and was preserved in 95% ethanol, and then transferred to −20 °C in laboratory for long-term storage at Nanjing Normal University (specimen voucher: NJNU-Nmad04; Hao Wang; 1216242141@qq.com). Total genomic DNA was extracted with standard phenol–chloroform methods (Sambrook et al. 1989). The complete mitochondrial genome sequencing and assembly were performed by Novogene (Beijing, China). The sequencing libraries with average insert sizes of approximately 300 bp were prepared, and then sequenced as 150 bp paired-end runs (about 15 Gb raw data) on the Illumina HiSeq 2500 platform (Illumina, San Diego, CA). De novo assemblies were conducted with Geneious 9.1.4 using the mitogenome of *Numenius phaeopus* (GenBank no. KP308149) as reference Map with default parameter settings (Kearse et al. 2012). The mitogenome were annotated by the MITOS Web server (Bernt et al. 2013), and then modified using BLAST alignment against the closely related species *N. phaeopus*.

The circular mitogenomes is 17,147 bp in length, consisting of 13 protein-coding genes, two ribosomal RNAs (12S rRNA and 16S rRNA), 22 transfer RNA genes, and a non-coding region, and all the genes are identified, without showing any structural rearrangement (GenBank no.: MW930394). The gene of ND6 and eight tRNA (tRNA^Gln^, tRNA^Ala^, tRNA^Asn^, tRNA^Cys^, tRNA^Tyr^, tRNA^Ser^, tRNA^Pro^, and tRNA^Glu^) are encoded on the light strand, whereas the other genes are located on the heavy strand. The overall nucleotide composition was A: 31.0%, T: 25.6%, C: 29.5%, and G: 13.9%. The total length of 13 protein coding genes is 11,393 bp accounting for 66.44% of the complete genome. The start codon ATG appeared in 10 PCGs, except for COI (GTG), ND3 (ATA), and ND6 (CTA). The use of stop codons is more diverse, relatively. Four stop codons (TAA, AGG, TAG, and AGA) and an incomplete stop codon (T–) were used.

The pairwise K2P (Kimura two-parameter) distance of mtDNA between *N. madagascariensis* and *N. tenuirostris* was 0.042. Phylogenetic reconstruction was performed using the maximum-likelihood method (ML) with the software of MEGA X (Kumar et al. 2018). The dataset of nearly complete mitogenome (15,885 bp) of 19 Scolopacidae species was used, and two species were used (*Larus brunnicephalus* JX155863, *Larus crassirostris* KM507782) as outgroups. ML phylogeny was inferred under the GTR+*G + I* model for 1000 bootstraps. The phylogenetic analysis (Figure 1) resolved great mitochondrial divergence within the Scolopacidae. This study expands our comprehension of the evolution of mitogenome in Scolopacidae.

**Figure 1.**
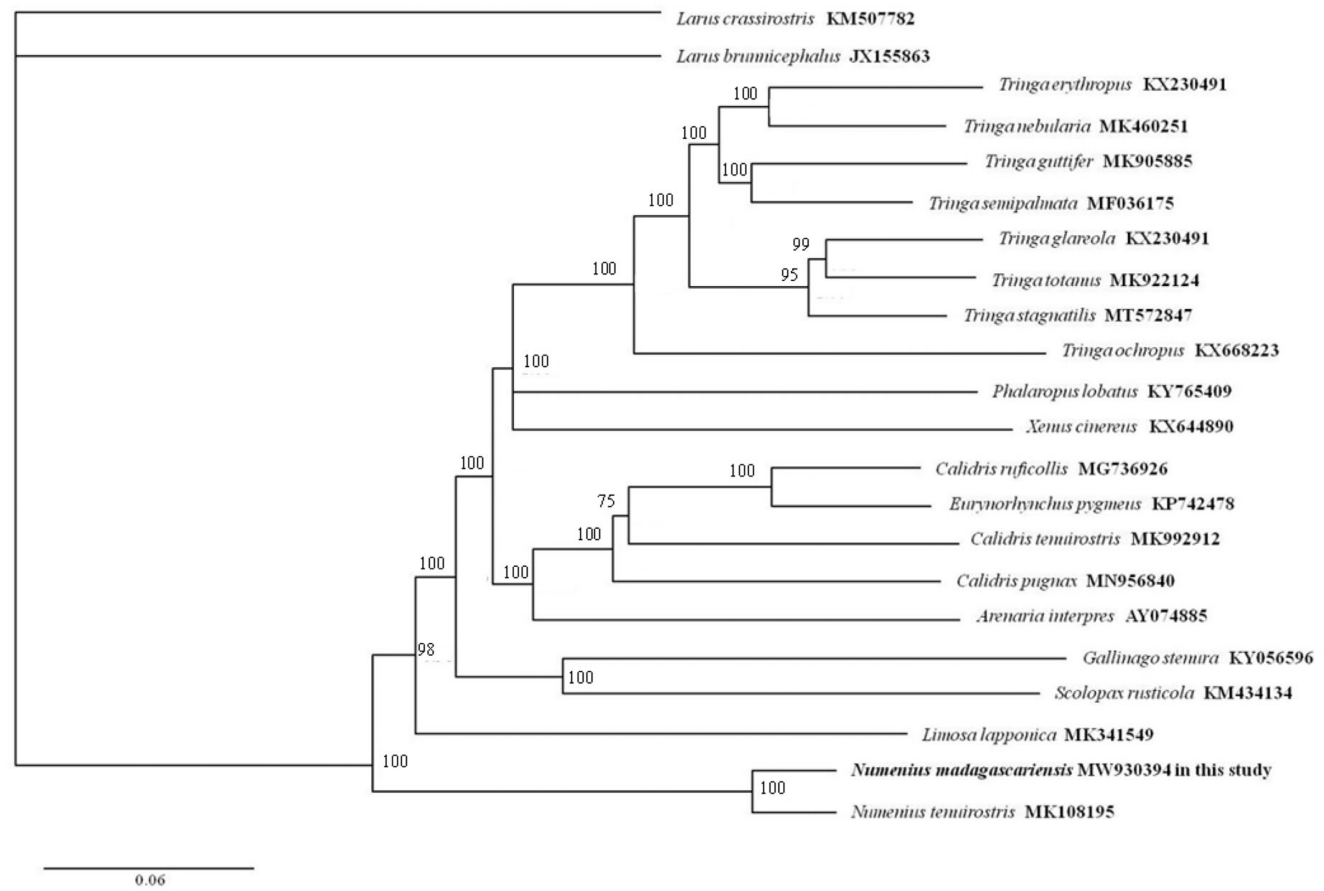
Phylogeny of *Numenius madagascariensis* and closely related 19 mitochondrial sequences constructed using the maximum-likelihood (ML) method by analyzing mitochondrial complete genome. Numbers above each branch are the ML bootstrap support.

## Data Availability

The genome sequence data that support the findings of this study are openly available in GenBank of NCBI at https://www.ncbi.nlm.nih.gov/ under the accession no. MW930394. The associated BioProject, SRA, and Bio-Sample numbers are PRJNA722761, SUB9495905, and SAMN18791592, respectively.
